# Comparing research recruitment strategies to prospectively identify patients presenting with breathlessness in primary care

**DOI:** 10.1038/s41533-022-00308-5

**Published:** 2022-11-09

**Authors:** Gillian Doe, Simon Wathall, Jill Clanchy, Sarah Edwards, Helen Evans, Michael C. Steiner, Rachael A. Evans

**Affiliations:** 1 Department of Respiratory Science, University of Leicester, Leicester, UK; 2 Clinical Trials Unit, Keele University, Newcastle-under-Lyme, UK; 3 Clinical Trials Unit, University of Leicester, Leicester, UK; 4 NIHR Biomedical Research Centre—Respiratory theme, University Hospitals of Leicester NHS Trust, Leicester, UK

**Keywords:** Clinical trial design, Outcomes research

## Abstract

Two recruitment strategies for research were compared to prospectively identify patients with breathlessness who are awaiting a diagnosis in primary care. The first method utilised searches of the electronic patient record (EPR), the second method involved an electronic template triggered during a consultation. Using an electronic template triggered at the point of consultation increased recruitment to prospective research approximately nine-fold compared with searching for symptom codes and study mailouts.

Patients living with breathlessness often seek help for the first time in primary care; around 4% of general practitioner (GP) consultations are due to breathlessness, and much of their management is undertaken in primary care^1^. This population commonly have underlying cardiorespiratory disease^2^, but there are significant delays in diagnosis and variation in clinical practice^3–5^. Successful recruitment to research trials in primary care is key to improving outcomes for adults presenting with breathlessness, but there are significant challenges, particularly with intervention trials^6^. A review of effective research recruitment in primary care indicates that the key elements include practitioner involvement, simple eligibility criteria, participant incentives and minimal impact on practitioner workload^7^.

We aimed to compare two prospective recruitment strategies for adults presenting with breathlessness for the first time in primary care using similar GP practices.

## Methods

### Recruitment strategies and participants

**Fig. 1:**
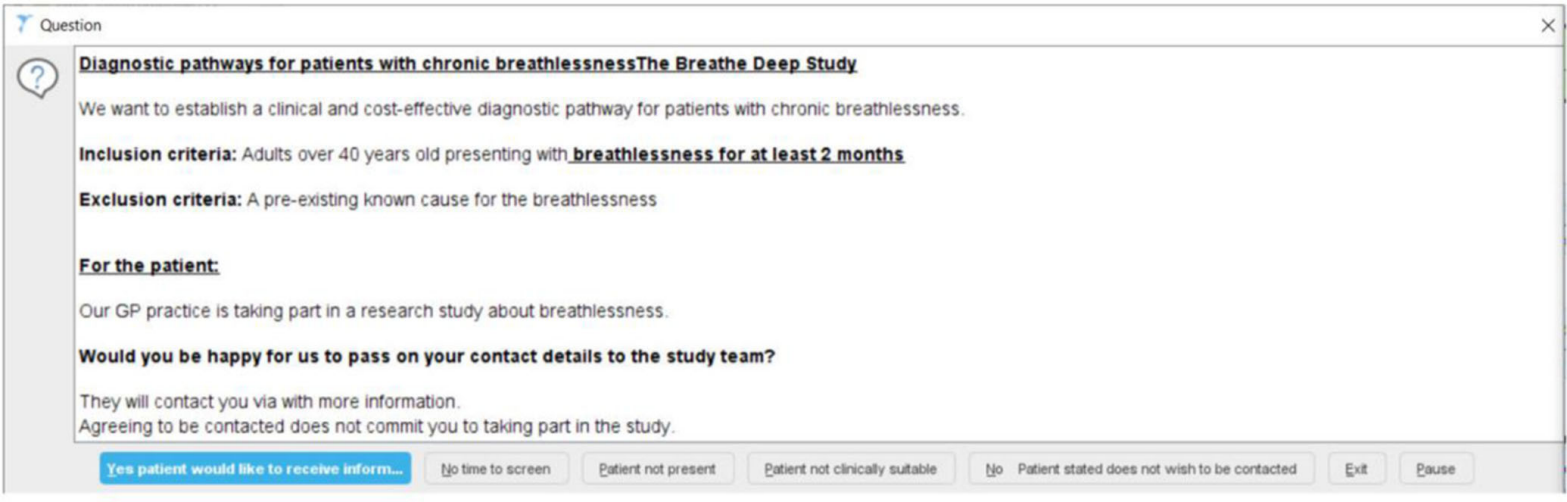
Opportunistic Approach (Strategy 2). Electronic template triggered on consultation with the GP.

Two different strategies were applied to prospectively identify and recruit patients presenting with breathlessness to GP practices in Leicestershire, UK. The first method (Strategy 1) for a primary care breathlessness cohort study used weekly searches for new breathlessness Read codes in the electronic patient record (EPR), followed by a mail out of study information to identified patients at 14 GP practices. The second method (Strategy 2) implemented an opportunist approach using an electronic template on the EPR, triggered at the point of consultation by either breathlessness-free text or Read codes at 10 GP practices^8^. The template (Fig. 1) summarised the study and eligibility criteria, prompting GPs to ask patients’ permission to be contacted by the study team. The wording for the electronic template to aid recruitment was developed by members of the patient and public involvement (PPI) group. The electronic template was designed to maximise identification of patients with specific eligibility; first or second presentation with breathlessness, over the age of 40 years old and without a pre-existing diagnosis (e.g. Chronic Obstructive Pulmonary Disease [COPD] and heart failure). The pop-up, therefore, did not trigger in certain patient records to avoid the unnecessary burden and filtering out by the clinician. The protocol for the electronic template is included in the supplementary information. Weekly reports of the patients identified were sent securely via nhs.net to the study team.

The electronic template was developed in partnership with Keele Clinical Trials Unit who supported the implementation of the electronic patient record system (SystmOne and Egton Medical Information Systems [EMIS]) for each practice. In addition to enhancing opportunistic recruitment, the template was embedded in the clinical care template for GP practices in the intervention with links to the next steps needed for the trial and links to best practice guidelines for the usual care GP practices.

### Research studies design

The studies using the different recruitment strategies were distinct in their purpose; one a cohort study and the second a feasibility cluster randomised controlled trial (cRCT). However, the participant eligibility and involvement were similar, requiring research visits of the same frequency, to the same location, and completion of the same outcome measures. The same Read codes were used for both strategies and are available in the supplementary information. The GP practices in both studies were a similar size from the same three clinical commissioning groups within the same county. The recruitment rate was compared at six months from each of the trial start dates.

Semi-structured interviews with patients and GP practice staff were performed, including experiences with the electronic template method, breathlessness and healthcare interactions. Interviews were audio-recorded, transcribed, coded and reviewed by the study team using thematic analysis^9^. Written informed consent was obtained from all participants recruited in both Strategy one and two. The study described in Strategy 2 was registered with ISRCTN (ID:14483247, date registered: 06/11/2019).

### Ethical approval

Research Ethics approval was provided by Wales Research Ethics Committee (REC) 7 (REC Reference 18/WA/0022) for Strategy 1 and Nottingham REC 1 (REC Reference: 19/EM/0201) for Strategy 2.

**Fig. 2:**
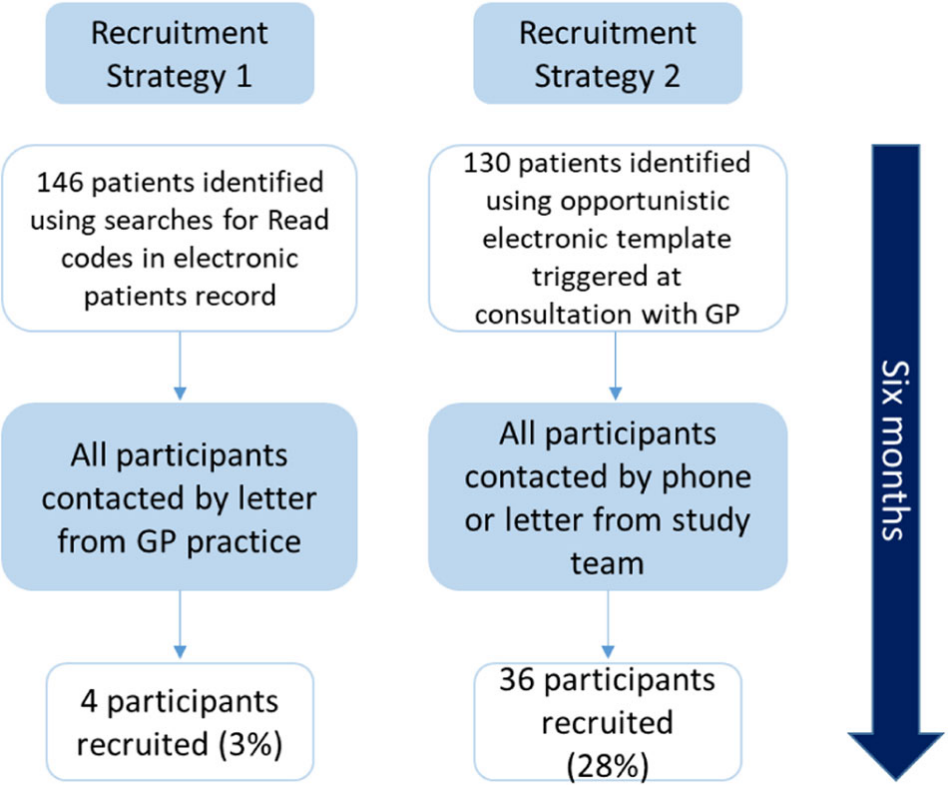
Recruitment flow diagram. A comparison of recruitment numbers between Strategy 1 and 2 over six months.

Over 6 months, more participants were identified and recruited using Strategy 2, 36/130 (28%) compared with Strategy 1, 4/146 (3%) participants (Fig. 2). The proportion of patients identified to the study team using Strategy 2 ranged from 6 to 14% of those where the template was triggered (our best estimate of the true denominator). In-depth review of the template activity in six of the GP practices showed the template was closed by clinicians without action for 33% (311/980) of patients and that there was no difference in frequency of triggering pre and post-March 2020 when the pandemic began.

Ten clinicians (nine GPs and one Respiratory Nurse) and seven administrative staff from each of the practices using the electronic template in Strategy 2 were interviewed to explore their views on the recruitment process, workload and study experience. Overall, GPs found the electronic template to be unobtrusive and helpful to have prompts, and patients were positive about receiving information about research from their GP (Table 1). One GP expressed finding the frequency of the template pop-up an annoyance during consultations.

**Table 1:** Healthcare practitioner and patient experience of recruitment at the time of presentation supported by an electronic ‘pop-up’ template (Strategy 2).

Quotes from GPs and practice staff describing the electronic template recruitment process
“Well, I have to say it’s been very unobtrusive hasn’t it. Because all that you’ve been asking us to do is ask the patient.” (I_BD03, Clinician)
“I think on SystmOne as soon as you type breathlessness all of the information comes up which is really great. I think it prompts people to think about the study and to think about, is this patient possibly suitable?” (I_BD04, Clinician)
“I think pretty good actually. I think I’ve found, because our role has just been to try and recruit, so it’s a fairly straightforward would you be interested or not?” (I_BD06, Clinician)
“Yeah, so they’ve found it useful as in they didn’t really have to think, it would pop up if the patient was eligible and then they only had to ask there. So that was useful. But once again since COVID, everybody’s breathless, so it popped up more times than it probably should have, because obviously more people are becoming more breathless with COVID and things like that. But before that I think it worked pretty well, because it’s just like a little reminder to the GPs to ask if they want to participate.” (I_BD07, Admin Staff)
“I think it was working quite well. And they do say, they will usually tell you if they don’t like something….Yeah, if it had come up on an inappropriate patient all the time, then they would say take this off, this is driving us mad or whatever. They would tell you yeah.” (I_BD08, Admin Staff)
“There’s a few GPs that get irritated by too many pop-ups, so I’ve had the odd comment about it. But I think that’s sometimes more a reflection of just the general stress and tiredness that everyone’s feeling at the moment more than anything.” (I_BD16, Admin Staff)
“…annoying to be honest, because I didn’t know what it was about, because we’re writing that so often that it just kept popping up.” (I_BD13, Clinician)

Quotes from patients describing their interaction with the GP about the study
“Yes basically I was struggling with my breathing and sort of got a chest infection. So I went down to the doctors and they asked me if I’d be interested in taking part in a breathlessness study. So I said yeah fine lovely. We can only improve with it, we can’t, we’re not going to go backwards, we can only go forwards with it” (BD29, patient, male)
“So then I went to the doctors. And it was the doctor who told me about it *[the research study]*.” (BD30, patient, male)
“I discussed that with my doctor. And at the time I think there was this survey going on, said do you mind going, me referring you to the survey so they can maybe check and see what’s happening, and how.” (BD17, patient, female)

Strategy 1 was limited by a lack of coding for breathlessness, and therefore searching for breathlessness-related Read codes did not yield enough eligible patients to contact, and once contacted were less likely to participate. GPs frequently code for diagnoses in primary care on the electronic patient record, previously using Read codes and more recently using SNOMED codes in the UK. GPs are less likely to code for symptoms such as breathlessness which are more commonly added as free text^10^, with the expectation that once a diagnosis is reached then the corresponding code will be added. The aim of coding is to provide a standardised vocabulary for clinicians to record patient findings^11^. However, clinical practice research datalink (CPRD) work has highlighted the challenges in using code lists in primary care to search for clinical features of interest, with inconsistent use of code labels^5,10^.

In contrast, the opportunistic use of an electronic ‘pop-up’ within a clinical consult in primary care has previously been used successfully to recruit to several trials for other symptom-based research and interventions, including musculoskeletal problems and back pain^12,13^ and appeared to increase recruitment of patients presenting with breathlessness in our data. During initial engagement and consultation with GPs and practice staff prior to the study set up some described ‘pop up fatigue’ for this kind of template, and the design was refined. However, our qualitative data demonstrate that the majority of practices found this to be an effective prompt with minimal disruption to the clinical consultation (Table 1). The careful consideration of the wording used at the point of template design, and consultation with our patient and public involvement (PPI) group, helped to ensure this was user-friendly and caused minimal disruption to the patient–clinician interaction.

The qualitative data from clinicians suggest that the impact of COVID-19 during the study period was perceived to increase the triggering of the electronic template, causing a problem for clinicians as more patients were presenting with breathlessness. This may have been perceived irritation during a time of high pressure, as review of the number of triggers at the GP practices demonstrated very little change in activity between March and August 2020. The way in which patients were accessing healthcare was significantly altered during this time, and the advantage of using the template was that it was always in use as a prompt whether consultations were face-to-face or by telephone.

There are limitations in our data as the two strategies were not compared within the same study design or period, and the GP practices taking part were not the same practices in the two studies. However, Strategy 2 was predominantly used during the COVID-19 pandemic in 2020, so our results may be underestimating recruitment in non-pandemic times. Coercion is an important ethical consideration when approaching patients for healthcare research via their usual health professional such as a GP^14^. To reduce this, the template used in Strategy 2 was only asking if patients could be contacted about a study for breathlessness, and not whether they were interested in taking part or consenting to take part in the research. Patients who gave permission were contacted by the study team with further information about the study and given time to make an informed decision. The key aspect of this work that made the electronic template an important asset was recruiting patients prospectively at the point of breathlessness presentation and in a primary care setting. There were no references in the interviews with patients to indicate they felt pressured to say yes when asked if they could be contacted regarding a research study.

An electronic template triggered at the point of consultation increased recruitment approximately nine-fold to prospective research compared with searching for symptom codes and study mailouts. Both healthcare professionals and patients were positive about the electronic template recruitment strategy. The electronic template is an effective method for researchers to consider maximising opportunistic patient recruitment and minimising the impact on clinician time in primary care.

### Reporting summary

Further information on research design is available in the Nature Research Reporting Summary linked to this article.

## Supplementary information

Supplemental Information

Reporting Summary

## Data Availability

The datasets generated and analysed during the current study are available from the corresponding author on reasonable request via email that is related to this work on recruitment strategies. The data generated relevant to study specific outcomes for each respective study described in this paper are still in the analysis and writing up stage and will not be available prior to publication of the respective study results.
