# Supplementary material for: Comparing research recruitment strategies to prospectively identify patients presenting with breathlessness in primary care
Source: NPJ Prim Care Respir Med. 2022 Nov 9;32:49. doi: 10.1038/s41533-022-00308-5 (PMC9646257; doi:10.1038/s41533-022-00308-5)
Supplement: Supplementary file 1 — Supplemental Information [file 41533_2022_308_MOESM1_ESM.pdf]

Supplementary Information

- Design flow for Strategy 2 Recruitment
- Read codes used for both Strategy 1 and 2

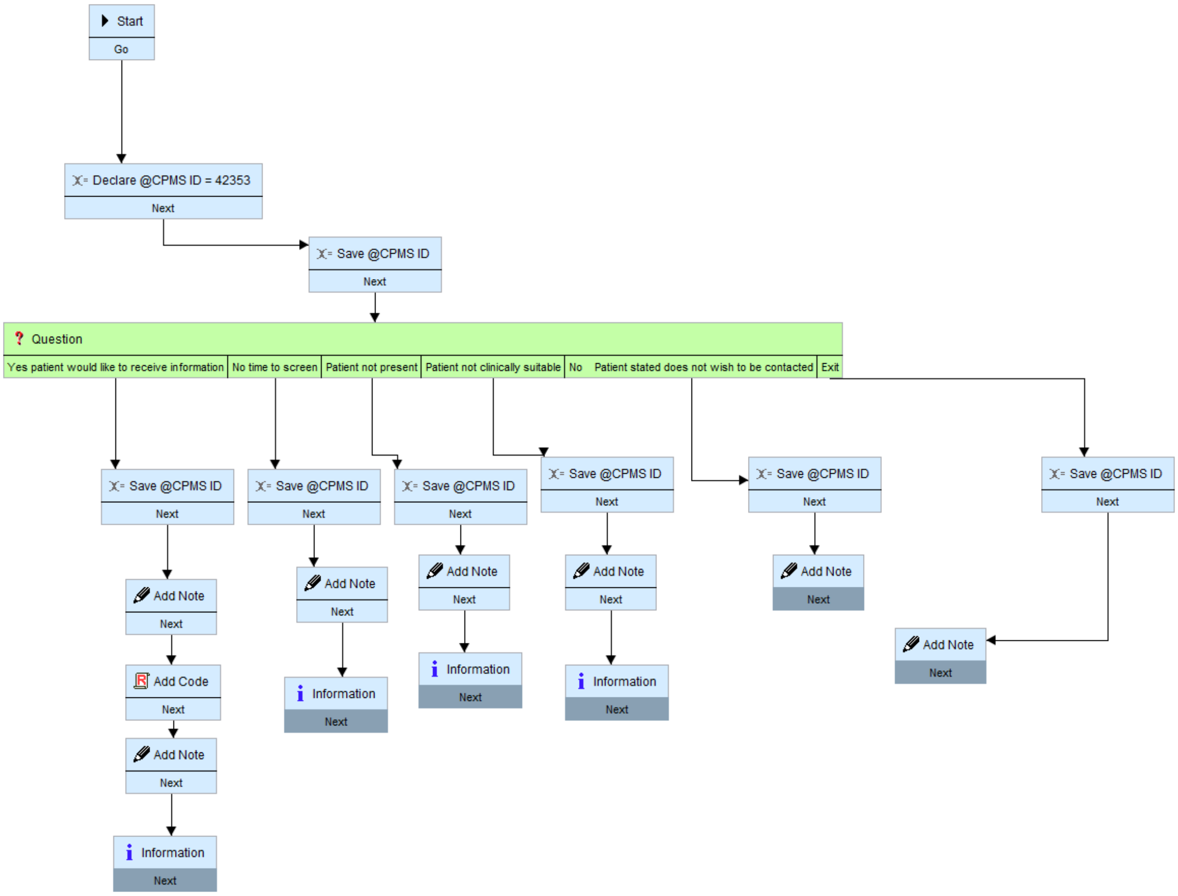

Trigger

- Can be triggered manually
- Can be triggered by free text: Breathless and SOB
- Automatically triggered when any of these codes (or their children) are entered:

[D]Breathlessness (XaBMU) (SNOMED: 267036007),

[D]Dyspnoea (R060A) (SNOMED: 267036007),

Breathless - moderate exertion (1732.) (SNOMED: 161939006),

Breathless - mild exertion (1733.) (SNOMED: 161940008),

Dyspnoea at rest (1734.) (SNOMED: 161941007),

Difficulty breathing (1738.) (SNOMED: 230145002),

Breathlessness NOS (173Z.) (SNOMED: 267036007),

[D]Orthopnoea (R0602) (SNOMED: 62744007),

[D]Shortness of breath (R0608) (SNOMED: 267036007),

Dyspnoea on exertion (X76Gz) (SNOMED: 60845006),

Breathlessness rating (X77QT) (SNOMED: 251896001),

Increasing breathlessness (Xa6a8) (SNOMED: 297216006),

Anxiety about breathlessness (Xaafv) (SNOMED: 702535006),

Breathlessness causing difficulty eating (XaagZ) (SNOMED: 702581006),

Breathless - strenuous exertion (XaIQ3) (SNOMED: 390871002),

MRC Breathlessness Scale: grade 1 (XaIUi) (SNOMED: 391120009),

MRC Breathlessness Scale: grade 2 (XaIUl) (SNOMED: 391123006),

MRC Breathlessness Scale: grade 3 (XaIUm) (SNOMED: 391124000),

MRC Breathlessness Scale: grade 4 (XaIUn) (SNOMED: 391125004),

MRC Breathlessness Scale: grade 5 (XaIUo) (SNOMED: 391126003),

Borg Breathlessness Score: 0 none at all (XaIvn) (SNOMED: 401275008),

Borg Breathlessness Score: 0.5 very, very slight (XaIvo) (SNOMED: 401323002),

Borg Breathlessness Score: 1 very slight (XaIvs) (SNOMED: 401279002),

Borg Breathlessness Score: 2 slight (XaIvt) (SNOMED: 401280004),

Borg Breathlessness Score: 3 moderate (XaIvu) (SNOMED: 401281000),

Borg Breathlessness Score: 4 somewhat severe (XaIvv) (SNOMED: 401282007),

Borg Breathlessness Score: 5 severe (XaIvy) (SNOMED: 401284008),

Borg Breathlessness Score: 6 severe (+) (XaIw0) (SNOMED: 401286005),

Borg Breathlessness Score: 7 very severe (XaIw5) (SNOMED: 401290007),

Borg Breathlessness Score: 8 very severe (+) (XaIw6) (SNOMED: 401291006),

Borg Breathlessness Score: 9 v, very severe (almost maximal) (XaIw7) (SNOMED: 401292004),

Borg Breathlessness Score: 10 maximal (XaIw8) (SNOMED: 401293009),

Dyspnoea (XE0qq) (SNOMED: 267036007),

Orthopnoea (XE0qr) (SNOMED: 62744007),

H/O: Breathlessness (Y2574),

Paroxysmal nocturnal dyspnoea (1736.) (SNOMED: 55442000),

O/E - dyspnoea (2322.) (SNOMED: 162890008),

Nocturnal dyspnoea (X76Gy) (SNOMED: 248548009),

Body mass airflow obstruct dyspnoea exercise capacity index (XaX8r) (SNOMED: 758801000000102),

Chronic respiratory disease questionnaire dyspnoea subscale (XaXfl) (SNOMED: 473339009),

Dyspnoea,obstruction, smoking, exacerbation frequency index (XaZZr) (SNOMED: 845231000000103),

Dyspnoea: no (Y09ae),

Dyspnoea Obstruction Smoking Status Exacerbation Frequency (DOSE) Score (Y0e6d),

Frequency nocturnal dyspnoea (YA297),

Dyspnoea trigger (YA298) or

Medical research council dyspnoea score (YA667)

Can only be triggered for:

[Role] GP Assistant

[Role] GP Associate

[Role] GP CMO

[Role] GP Locum

[Role] GP Partner

[Role] GP Registrar

[Role] GP Retainer

[Role] GP Sole Practitioner

[Role] General Medical Practitioner

[Role] Practice Manager

[Role] System Administrator

Filter

Only applies to patients over 40 years

Only applies to patients in the Breathe Deep - Patient eligible for protocol report

Exclusions

____	Mandatory In
- - - -	Optional In
....	Not In

Breathe Deep - Patient eligible for protocol
Research / Breathlessness

Matching to GMS, Applied, Private, Temporary, Other, Blank, Walk-in, Online User patients

NOT IN → **Breathe Deep - Exc 4 - Clinical exclusions**
Research / Breathlessness

Has a Read code in...Codes and Children:
Rheumatic left ventricular failure (G1yz1) (SNOMED: 43736008)
Heart failure (G58..) (SNOMED: 84114007)
Obliterating fibrous bronchiolitis (H0614) (SNOMED: 40100001)
Chronic obstructive lung disease (H3...) (SNOMED: 13645005)
Chronic emphysema due to chemical fumes (H4640) (SNOMED: 196026004)
Interstitial lung disease NEC (H58y3) (SNOMED: 233703007)
[X]Other specified chronic obstructive pulmonary disease (Hyu31) (SNOMED: 13645005)
Occupational chronic bronchitis (X101j) (SNOMED: 49691004)
Byssinosis grade 3 (X101k) (SNOMED: 233672007)
Drug-induced bronchiolitis obliterans (X101m) (SNOMED: 233673002)
Pulmonary emphysema (X101n) (SNOMED: 87433001)
Pulmonary emphysema in alpha-1 PI deficiency (X101o) (SNOMED: 233674008)
Toxic emphysema (X101p) (SNOMED: 233675009)
Congenital lobar emphysema (X101q) (SNOMED: 66987001)
Scar emphysema (X101r) (SNOMED: 233677001)
Heart failure as a complication of care (X202k) (SNOMED: 233924009)
Right ventricular failure (X202l) (SNOMED: 367363000)
Biventricular failure (XE0V8) (SNOMED: 92506005)
Heart failure NOS (XE0V9) (SNOMED: 84114007)
Purulent chronic bronchitis (XE0YM) (SNOMED: 84409004)
Bullous emphysema with collapse (XE0YN) (SNOMED: 266355005)
Atrophic (senile) emphysema (XE0YO) (SNOMED: 266356006)
Other emphysema NOS (XE0YP) (SNOMED: 87433001)
Left ventricular failure (XE2QG) (SNOMED: 85232009)
Impaired left ventricular function (XM1Qn) (SNOMED: 275514001)
Late effects of infectious disease NOS (XaEGY) (SNOMED: 720349002)

Mild chronic obstructive pulmonary disease (XaEIV) (SNOMED: 313296004)
Moderate chronic obstructive pulmonary disease (XaEIW) (SNOMED: 313297008)
Severe chronic obstructive pulmonary disease (XaEIY) (SNOMED: 313299006)
End stage chronic obstructive airways disease (XaIND) (SNOMED: 135836000)
Interstitial pulmonary emphysema (XaIQg) (SNOMED: 77690003)
New York Heart Association classification - class I (XaJ9G) (SNOMED: 420300004)
New York Heart Association classification - class II (XaJ9H) (SNOMED: 421704003)
New York Heart Association classification - class III (XaJ9I) (SNOMED: 420913000)
New York Heart Association classification - class IV (XaJ9J) (SNOMED: 422293003)
Chronic obstructive pulmonary disease finding (XaK8Q) (SNOMED: 13645005)
Very severe chronic obstructive pulmonary disease (XaN4a) (SNOMED: 293991000000106)
Congestive heart failure due to valvular disease (XaO5n) (SNOMED: 426611007)
Heart failure with normal ejection fraction (XaWyi) (SNOMED: 446221000)
Eosinophilic bronchitis (Xaa7C) (SNOMED: 866901000000103)
Asthma-chronic obstructive pulmonary

Heart failure with reduced ejection fraction (XafeB) (SNOMED: 703272007)

Date of Read code before 184 days ago

Matching to GMS, Applied, Private, Temporary, Other, Blank, Walk-in, Online User patients

NOT IN → **Breathe Deep - Exc 1 - Patients to be sent an invitation**
Research / Breathlessness

Has a Invitation to participate in clinical trial = 42353.0 CPMS

Matching to GMS, Applied, Private, Temporary, Other, Blank, Walk-in, Online User patients

NOT IN → **Breathe Deep - Exc 3 - GP screened ineligible**
Research / Breathlessness

Has a Does not meet eligibility criteria for clinical trial = 42353.0 CPMS

Matching to GMS, Applied, Private, Temporary, Other, Blank, Walk-in, Online User patients

NOT IN → **Breathe Deep - Exc 2 - Declined invite**
Research / Breathlessness

Has a Declined invitation to participate in research study = 42353.0 CPMS

Matching to GMS, Applied, Private, Temporary, Other, Blank, Walk-in, Online User patients
