# Supplementary material for: Comparing research recruitment strategies to prospectively identify patients presenting with breathlessness in primary care
Source: NPJ Prim Care Respir Med. 2022 Nov 9;32:49. doi: 10.1038/s41533-022-00308-5 (PMC9646257; doi:10.1038/s41533-022-00308-5)
Supplement: Supplementary file 2 — Reporting Summary [file 41533_2022_308_MOESM2_ESM.pdf]

nature portfolio

Corresponding author(s): Dr Rachael Evans

Last updated by author(s): Aug 1, 2022

Reporting Summary

Nature Portfolio wishes to improve the reproducibility of the work that we publish. This form provides structure for consistency and transparency in reporting. For further information on Nature Portfolio policies, see our Editorial Policies and the Editorial Policy Checklist.

Statistics

For all statistical analyses, confirm that the following items are present in the figure legend, table legend, main text, or Methods section.

n/a	Confirmed	
☐	☒	The exact sample size (*n*) for each experimental group/condition, given as a discrete number and unit of measurement
☐	☒	A statement on whether measurements were taken from distinct samples or whether the same sample was measured repeatedly
☒	☐	The statistical test(s) used AND whether they are one- or two-sided<br>*Only common tests should be described solely by name; describe more complex techniques in the Methods section.*
☒	☐	A description of all covariates tested
☒	☐	A description of any assumptions or corrections, such as tests of normality and adjustment for multiple comparisons
☒	☐	A full description of the statistical parameters including central tendency (e.g. means) or other basic estimates (e.g. regression coefficient) AND variation (e.g. standard deviation) or associated estimates of uncertainty (e.g. confidence intervals)
☒	☐	For null hypothesis testing, the test statistic (e.g. *F*, *t*, *r*) with confidence intervals, effect sizes, degrees of freedom and *P* value noted<br>*Give P values as exact values whenever suitable.*
☒	☐	For Bayesian analysis, information on the choice of priors and Markov chain Monte Carlo settings
☒	☐	For hierarchical and complex designs, identification of the appropriate level for tests and full reporting of outcomes
☒	☐	Estimates of effect sizes (e.g. Cohen's *d*, Pearson's *r*), indicating how they were calculated

Our web collection on statistics for biologists contains articles on many of the points above.

Software and code

Policy information about availability of computer code

Data collection	n/a
Data analysis	n/a

For manuscripts utilizing custom algorithms or software that are central to the research but not yet described in published literature, software must be made available to editors and reviewers. We strongly encourage code deposition in a community repository (e.g. GitHub). See the Nature Portfolio guidelines for submitting code & software for further information.

Data

Policy information about availability of data

All manuscripts must include a data availability statement. This statement should provide the following information, where applicable:

- Accession codes, unique identifiers, or web links for publicly available datasets
- A description of any restrictions on data availability
- For clinical datasets or third party data, please ensure that the statement adheres to our policy

The data sets generated and analysed during the current study are available from the corresponding author on reasonable request.

Human research participants

Policy information about studies involving human research participants and Sex and Gender in Research.

Reporting on sex and gender	n/a<br>Overall participant recruitment reported only.
Population characteristics	n/a<br>Recruitment rate only was applicable to this manuscript.
Recruitment	The manuscript is all related to Recruitment strategy (a comparison of two strategies).
Ethics oversight	Research Ethics approval was provided by Wales Research Ethics Committee (REC) 7 (REC Reference 18/WA/0022) for Strategy 1 and Nottingham REC 1 (REC Reference: 19/EM/0201) for Strategy 2.

Note that full information on the approval of the study protocol must also be provided in the manuscript.

Field-specific reporting

Please select the one below that is the best fit for your research. If you are not sure, read the appropriate sections before making your selection.

☐ Life sciences ☒ Behavioural & social sciences ☐ Ecological, evolutionary & environmental sciences

For a reference copy of the document with all sections, see nature.com/documents/nr-reporting-summary-flat.pdf

Behavioural & social sciences study design

All studies must disclose on these points even when the disclosure is negative.

Study description	Two recruitment strategies for research were compared to prospectively identify patients with breathlessness who are awaiting a diagnosis in primary care. The first method utilised searches of the electronic patient record (EPR), the second method involved an electronic template triggered during a consultation. Recruitment rate was recorded for each strategy.<br>Semi-structured interviews with patients and GP practice staff were performed as part of the one of the studies described in this manuscript, including experiences of the electronic template method, breathlessness and healthcare interactions.
Research sample	The research sample were patients presenting to their GP with breathlessness. Participants were recruited to there respective studies (one cohort study and one cluster randomised controlled trial) as per study protocols and the recruitment strategies employed by the two studies were compared in this manuscript.
Sampling strategy	Two different strategies were applied to prospectively identify and recruit patients presenting with breathlessness to GP practices in Leicestershire, UK. The first method (Strategy 1) for a primary care breathlessness cohort study used weekly searches for new breathlessness Read codes in the electronic patient record (EPR), followed by a mail out of study information to identified patients at 14 GP practices. The second method (Strategy 2) implemented an opportunist approach using an electronic template on the EPR, triggered at the point of consultation by either breathlessness free text or Read codes at 10 GP practices.
Data collection	For this manuscript data relating to recruitment rate was recorded.<br>Qualitative data relating to the use of the second recruitment strategy was also collected by interview. Interviews were audio-recorded, transcribed, coded and reviewed by the study team using thematic analysis.
Timing	The recruitment rate was compared at six months from each of the trial start dates. Strategy one recruited patients from July 2018 and Strategy two recruited patients from October 2019.
Data exclusions	Data re: recruitment rate was included for the first six months of recruitment for both strategies to allow comparison. Strategy two did continue recruitment for 17 months.
Non-participation	n/a
Randomization	Participants were taking part in separate research studies. It was only the recruitment strategies used that have been compared.

Reporting for specific materials, systems and methods

We require information from authors about some types of materials, experimental systems and methods used in many studies. Here, indicate whether each material, system or method listed is relevant to your study. If you are not sure if a list item applies to your research, read the appropriate section before selecting a response.

Materials & experimental systems

n/a	Involved in the study
☒	☐ Antibodies
☒	☐ Eukaryotic cell lines
☒	☐ Palaeontology and archaeology
☒	☐ Animals and other organisms
☒	☐ Clinical data
☒	☐ Dual use research of concern

Methods

n/a	Involved in the study
☒	☐ ChIP-seq
☒	☐ Flow cytometry
☒	☐ MRI-based neuroimaging
